# Thermal Load Model of a Proportional Solenoid Valve Based on Random Load Conditions

**DOI:** 10.3390/s23239474

**Published:** 2023-11-28

**Authors:** Chenyu Liu, Anlin Wang, Xiaotian Li, Xiaoxiang Li

**Affiliations:** School of Mechanical Engineering, Tongji University, Shanghai 201804, China; wanganlin@tongji.edu.cn (A.W.); lixiaotian@tongji.edu.cn (X.L.); lixiaoxiang@tongji.edu.cn (X.L.)

**Keywords:** random load, proportional solenoid valve, Kalman filtering, k-means clustering, thermal load model

## Abstract

Drastic changes in the random load of an electromechanical system bring about a reliability problem for the proportional solenoid valve based on a thermal effect. In order to accurately and effectively express the thermal load of a proportional solenoid valve under random load conditions and to meet the requirements of online acquisition, adaptive anomaly detection, and the missing substitution of thermal load data, a thermal load prediction model based on the Kalman filter algorithm is proposed. Taking the compound operation process of an excavator as the object and based on the field testing of an excavator and the independent testing experiment of a proportional solenoid valve in a non-installed state, a method of obtaining historical samples of the proportional solenoid valve’s power and thermal load is given. The k-means clustering algorithm is used to cluster the historical samples of the power and thermal load corresponding to the working posture of a multi-tool excavator. The Grubbs criterion is used to eliminate the outliers in the clustering samples, and unbiased estimation is performed on the clustering samples to obtain the prediction model. The results show that the cross-validation of the sample data under the specific sample characteristics of the thermal load model was carried out. Compared with other methods, the prediction accuracy of the thermal load model based on the Kalman filter is higher, the adaptability is strong, and the maximum prediction deviation percentage is stable within 5%. This study has value as a reference for random cycle thermal load analyses of low-frequency electromechanical products.

## 1. Introduction

In the control systems of construction machinery, a proportional solenoid valve is often used as a pilot valve to achieve the pilot pressure control of multi-way valves and the inclination control of variable pumps. Due to its simple structure, ease of integration, suitability for mass production, and ability to make the overall structural design and layout of the control system’s hardware module more flexible, this approach is being more widely used in many engineering fields, including engineering machinery, automobiles, and aviation [1,2].

In the process of cyclic operation, the engineering machinery, represented here by an excavator, operates under complex working conditions and severe load changes. The dynamic process of random load fluctuations in the main electromechanical components of the proportional solenoid valve and other control systems is mainly determined by the working mode of the excavator, the structural parameters of the excavator, and the random factors contained in the working medium, and it is also related to the operating habits of the operator [3]. The pressure load of the proportional solenoid valve has the characteristics of being non-stationary, random, and cyclic. From the pressure load to the heat load, the current in the solenoid valve coil causes energy consumption, and the energy is dissipated in the form of heat, resulting in temperature increases inside and outside the solenoid valve. Due to the time inertia, the temperature affects the linear proportional input and output characteristics of the solenoid valve, the slide valve’s passability, and the life of the solenoid valve over a continuous and long time domain. By studying the input–output mapping relationship of the proportional solenoid valve during the excavator operation, the heat load characteristics of the solenoid valve during the working process are analyzed and a heat load model that can characterize the thermodynamic performance of the solenoid valve is constructed.

Accurate and effective solenoid valve energy consumption measurement data with a certain number of scales form the basis for effectively implementing the thermal load control of the electromechanical components in the excavator control system to ensure heat resistance and reliability. Being affected by adverse environmental conditions such as high temperatures, dust, and humidity, as well as adverse working conditions such as voltage fluctuations and electromagnetic interference, the energy consumption data provided by the measurement equipment are outside the normal fluctuation range, meaning that problems such as values exceeding the normal range and data loss will occur from time to time. Therefore, it is necessary to detect the energy consumption data in real-time, find abnormal data, fill the missing data gaps, and ensure the correct implementation of the thermal load control strategy. Most of the traditional energy consumption data detection methods lack adaptive ability, data filling is mostly implemented offline through linear interpolation, the data errors are large, and the real-time performance is low, making it difficult to meet the needs of industrial intelligent applications [4]. With the development of artificial intelligence technology and the emergence of the era of big data, the traditional symbolic computing, statistical, and sampling methods are gradually being replaced by machine learning, deep neural networks, and other related theoretical methods with high fuzzy information processing ability and abstract ability for uncertain problems. Such intelligent methods are widely used in modeling, data collection, analyses, and prediction [5,6].

In order to solve the problem of the statistical method of power load forecasting’s long calculation time for parameter identification, researchers such as Adebunmi [7] adopted a neural fuzzy model. The adaptive neuro-fuzzy inference system (ANFIS), artificial neural network (ANN), and multiple linear regression (MLR) models were simulated in the MATLAB environment, and their output results were compared using the root mean square error (RMSE) and mean absolute error (MAE). The ANFIS model is superior to other models, having the smallest RMSE and MAE error values. In order to solve the problems of the limited modeling ability and large computational workload, Hambali [8] proposed an artificial neural network for power load forecasting, with applications in data mining algorithms to predict power loads. Two ANN algorithms (MLP and RBF) and an SMO algorithm were used and compared. The results showed that the accuracy of the multi-layer perceptron model (MLP) is 86% with a mean absolute error (MAE) of 0.016, the accuracy of the radial basis function (RBF) is 76% with an MAE of 0.030, and the accuracy of the sequential minimum optimization (SMO) model is 85% with an MAE of 0.090, indicating that the power load forecasting level is promising. Bendaoud [9] proposed an innovative load forecasting method using the load profile (LP). Firstly, power consumption is analyzed to detect the different factors that affect the demand. Then, the fluctuation of the seasonal data is applied through the hourly temperature curve. LP-based prediction uses three levels of LP propagation. The short-term and medium-term load forecasting models are developed using a variety of artificial intelligence technologies. Short-term and mid-term load forecasting models were developed using multiple artificial intelligence techniques. Among them, a two-dimensional convolutional neural network (CNN) was used for the first time in load forecasting. The resulting prediction accuracy values of both the artificial intelligence (AI)-based and LP-based models were high, producing (MAPE = 0.80%, RMSE = 75.57 MW, Willmott’s Index (WI) = 0.99) for the two-dimensional CNN. Keynia F [10] proposed a composite method based on multi-layer perceptron neural network and optimization technology to solve the MTLF problem when solving the non-stationary, volatile, and non-linear characteristics of medium-term load signals and many input variables and relative parameters that affect the load mode. The method has an optimal training algorithm composed of two search algorithms of particle swarm optimization and improved ant lion optimizer and multi-layer perceptron neural network. The accuracy of the proposed prediction method is extensively evaluated based on several benchmark data sets.

**Figure 1:**
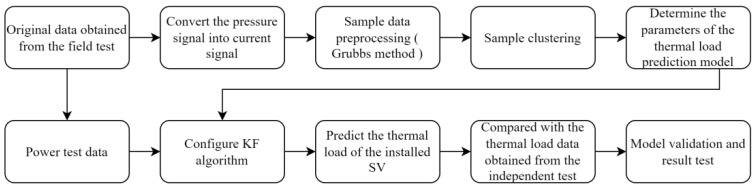
The flow chart of the method proposed in this paper.

Therefore, this paper proposes a thermal load prediction model of a solenoid valve based on the Kalman filter algorithm. The parameters of the model are determined by off-line statistical historical samples. The prediction calculation only depends on a small amount of data under certain working conditions. The calculation rules are simple and non-iterative. It can meet the needs of adaptive anomaly detection and missing filling in real time for the measurement data of the thermal load energy consumption of a solenoid valve in the random and changeable scenario of the composite operation load of construction machinery at low costs. The flow chart of the method proposed in this paper is shown in Figure 1.

## 2. Data Sample Collection and Sample Pretreatment Based on Field Test

### 2.1. Excavator Field Test

**Figure 2:**
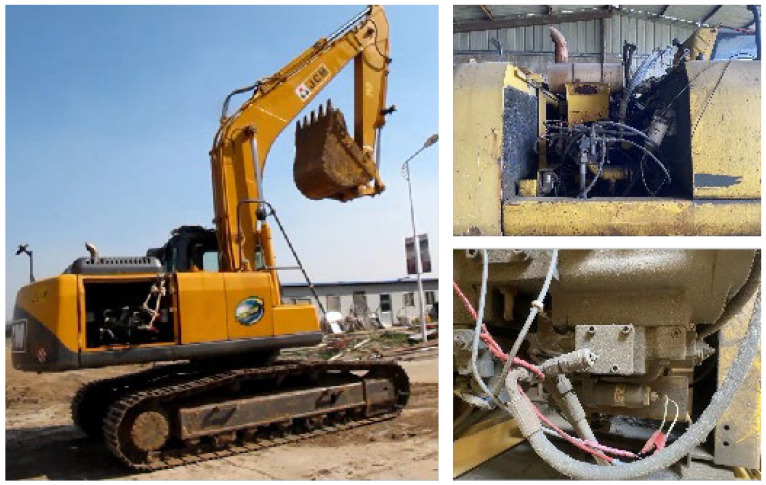
Field test environment and objects.

In order to truly reflect the performance of the proportional solenoid valve in the installed state and obtain real, reliable, and effective data samples, the excavator field test was adopted to obtain the original data samples. Under the condition of the on-site operation of the excavator, the parameters characterizing the dynamic traction performance of the excavator were directly measured and recorded. The field test took a 20 t medium-sized hydraulic excavator as the object [11]. The engine power was 112 kW, the standard bucket capacity was 1.2 m^3^, the rotation speed was 11.9 r/min, the walking speed was 3.9/5.6 km/h, the digging force of the bucket rod was 92.5 kN, the maximum digging width was 9839 mm, the maximum digging depth was 6590 mm, and the climbing ability was 70% (35 degrees). The field test environment and objects are shown in Figure 2. The test was carried out in the process of excavator composite operation. The composite operation process includes several common actions of the excavator, such as high-speed and low-speed walking, no-load and full-load rotation, separate excavation of each cylinder, and composite excavation. By reasonably arranging sensor measuring points on the platform, the pressure, flow rate, and system oil temperature of the proportional solenoid valve in the actual operation process of the excavator were obtained in real-time, synchronously and through multiple channels.

### 2.2. Data Sample Acquisition

**Figure 3:**
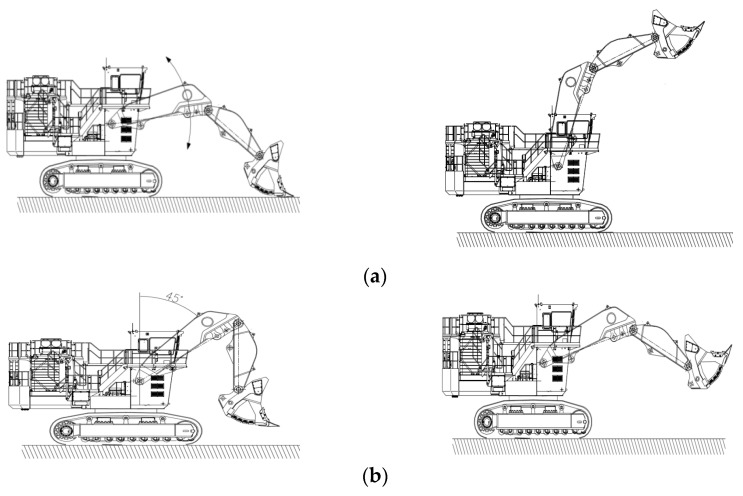

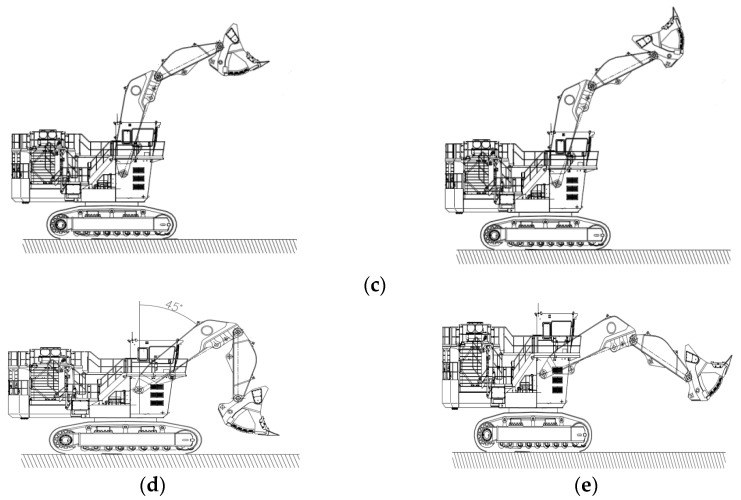
Typical single action posture of excavator. (**a**) Single action of boom; (**b**) Single action of bucket rod; (**c**) Single action of bucket; (**d**) Single action of walking; (**e**) Single action of turning.

A hydraulic excavator is a machine with severe load change. In the process of excavation, lifting, rotation, and unloading, its load size and intensity of fluctuation are very different, and each process is carried out repeatedly. There is a certain relationship between the change in excavator working load and the driver’s control tools, which is reflected in the dynamic test data of each measured parameter. The length of the test sample is very short, and it is necessary to repeat the test several times to obtain the overall sample of the test. The excavation process is more random, and it is impossible to determine the nature of stable data even if the test conditions are strictly controlled. In order to minimize the random interference of field tests and improve the operability of the data, several typical single-action conditions of the hydraulic excavator were designed, including single boom action, single bucket rod action, single bucket action, single walking action and single turning action. Figure 3 is a typical single-action posture diagram of excavator operation.

**Figure 4:**
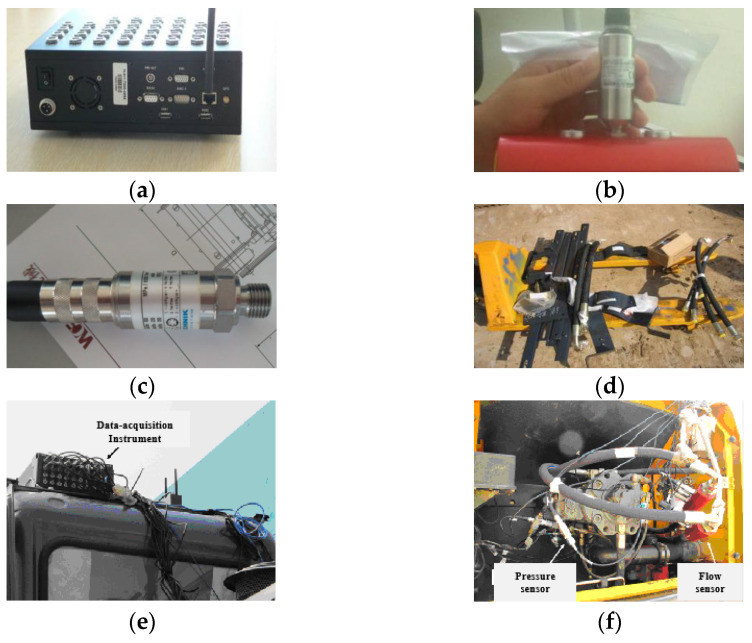

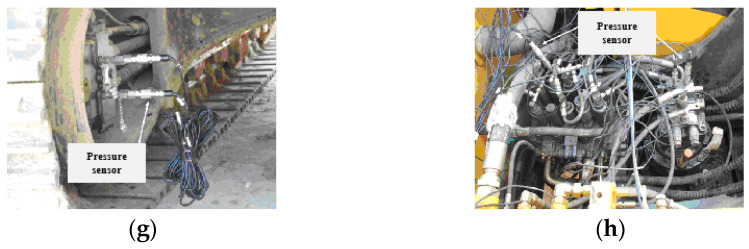
Site layout of sensor. (**a**) Data acquisition instrument; (**b**) Flow sensor; (**c**) Pressure sensor; (**d**) Test ready mounting; (**e**) Vehicle data acquisition instrument; (**f**) Pressure sensor and flow sensor of main pump; (**g**) Travel motor pressure sensor; (**h**) Rotary motor and main valve pressure sensor.

The main test instruments of the field test are the data acquisition instrument developed by the INTEST company, the flow sensor of the HYDAC company and the pressure sensor, notebook computer, and other auxiliary equipment of HYDROTECHNIK company. Figure 4 shows the main equipment and the installation position of the sensor. The sensor and data acquisition instrument were connected to the computer through the wireless router using the online measurement method. The online transmission of the measured data was carried out while the experimental measurement of the prototype was carried out, and the data collected on the spot were stored, displayed, and processed in real time by the computer. In the following text, only the measured data samples of proportional solenoid valves related to the research content of this paper are highlighted and processed.

During the field test, the original sampling data were the value of the working port pressure *p_A_* of the proportional solenoid valve. The solenoid valve is a proportional electromagnetic pilot pressure-reducing valve with a basin-type pull-in structure. The output electromagnetic thrust working at a rated current of 700 mA is between 20 and 30 N, and the control chamber pressure is about 20 bar. According to the structure and working mechanism of the solenoid valve, the control chamber pressure is the sum of the electromagnetic force *F_EM_* on the armature, the reset spring force *F_S_*, and the damping force *F_D_*, as expressed in Equation (1):

(1) $$\frac{\pi d^{2}p_{A}}{4}=F_{EM}-F_{S}-F_{D}$$

**Figure 5:**
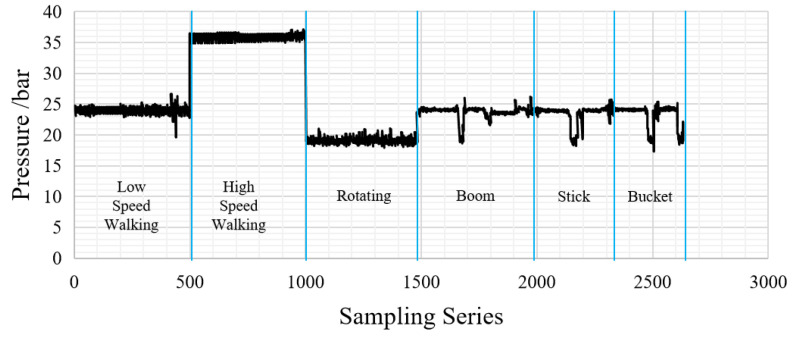
Pressure load sample sequence of the proportional solenoid valve.

The temperature of the electromagnetic coil rises due to the thermal effect during the operation, and the coil resistance increases with the temperature rise. The measured working pressure *p_A_* should be linearly proportional to the input current in the control loop, where the feedback loop in the control system performs feedback regulation on the input. Therefore, the linear mapping relationship between input and output can be constructed, and the measured pressure data can be converted into the coil current data, which are not easy to measure directly in the installed state for the thermal load prediction calculation in the subsequent research. Figure 5 shows the sample sequence of the pressure load of the proportional solenoid valve collected in the field test. It can be seen that the pressure load of the proportional solenoid valve under different operating modes had strong fluctuation randomness and a wide range of random sources. The main factors affecting the operation were the start and stop of the action of the operating mechanism, the reversing of the hydraulic cylinder, and so on.

**Figure 6:**
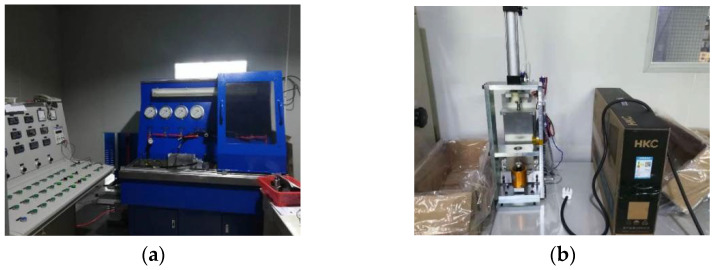
Independent test equipment of proportional solenoid valve in non-installed state. (**a**) Hydraulic pressure test platform; (**b**) Electromagnetic force test platform.

**Figure 7:**
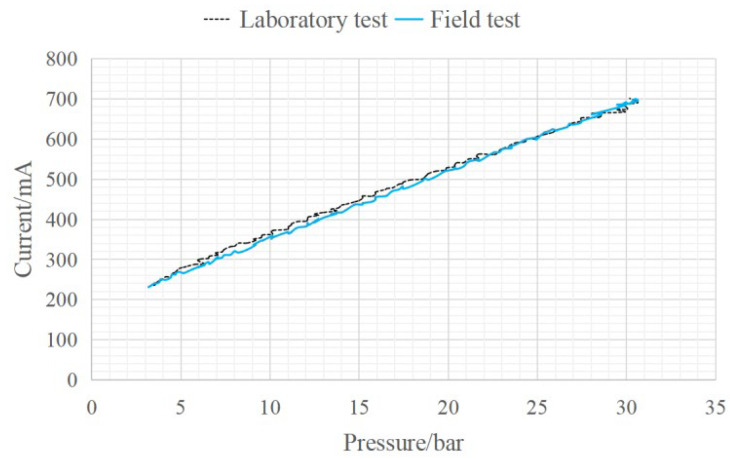
Input–output relationship of solenoid valve.

It can be determined from the product characteristics of the proportional solenoid valve that the current directly loaded on the electromagnetic coil has a linear proportional relationship with the electromagnetic force. Through the independent test of the proportional solenoid valve in the non-installed state, the fuzzy parameter information and uncertainty caused by the spring force and damping force during the force conduction process on the spool can be calibrated. The independent test includes the input–output test of the solenoid valve and the current–electromagnetic force test. The test equipment is shown in Figure 6. The input–output mapping relationship of the solenoid valve based on the independent test and field test is expressed by Equation (2). Considering that the installed position of the proportional solenoid valve spool is the main pump pilot port, the current measured in the field test is the current read in the controller. Due to the influence of the controller error and other unknown interference factors, the actual current in the proportional solenoid valve coil and the current read in the controller had a small value error within the acceptable range of the system. Figure 7 shows the comparison of the input and output mapping relationship of the solenoid valve based on the independent test experiment and the field test.

(2) $$\left\{ \begin{array}{l} P_{A}=0.0589\cdot I_{L}-10.892\cdots \cdots Laboratory\;test\\ P_{A}=0.0591\cdot I_{F}-10.751\cdots \cdots Field\;test \end{array}\right.$$

Here, *I_L_* and *I_F_* stand for the respective quantities of electric current in the laboratory and field tests.

### 2.3. Sample Pretreatment Method

In the actual operation scene of the excavator, the compound operation process is composed of typical single actions, and the samples of multiple operation modes are often mixed. Before an unbiased estimation is carried out to determine the parameters of the prediction model, it is necessary to cluster the samples to achieve preprocessing, and correspond the sample data with different tooling movements, or different operating modes of the excavator, in order to obtain the correct model parameters. The labeled information is often unknown from the data samples collected during the compound operation, which provides the basis for further data analysis. The clustering method reveals the intrinsic properties and laws of the data by learning the unlabeled samples [12]. The clustering method can be used as a separate process to find the intrinsic distribution structure of the data, and can also be used as a precursor process for other computing tasks. In this paper, the k-means clustering algorithm is adopted. Its advantages are a simple principle, easy implementation, fast convergence speed, better clustering effect, and strong interpretability of the algorithm. The main parameter to be adjusted is only the number of clusters k. The k-means clustering algorithm adopts a greedy strategy and approximates Equation (3) via iterative optimization.

(3) $$E=\sum_{i=1}^{k}\sum_{x\in C_{i}}||x-\mu_{i}|{|}_{2}^{2}$$

Here, *x* is the sample vector, *μ* is the mean vector of the cluster obtained by clustering, *C_i_* is the cluster divided by clustering, and *E* is the minimum square error.

After converting the pressure sample into the current sample, the clustering process of the new sample is as follows:

(1) The power-heat load two-dimensional space is constructed, and the power samples and heat load samples collected at the same or similar time are combined and mapped into coordinate points in the two-dimensional space.
(2) All the coordinate points in the two-dimensional space are randomly classified into several coordinate groups, and the arithmetic mean values of power and thermal load in each coordinate group are calculated respectively, and the obtained arithmetic mean value is taken as the center point coordinate of the coordinate group.
(3) Calculate the Euler distance from all coordinate points to the center point of each coordinate group in two-dimensional space, and re-divide the coordinate group to which the coordinate points belong according to the principle of minimum distance.
(4) Calculate the center point of the new coordinate group.
(5) Repeat (3)~(4) until the composition of each coordinate group is no longer changed.

The abnormal data that may be contained in the actual sampling samples cannot be screened out by the clustering process. Their existence will affect the unbiased estimation of the parameters of the thermal load prediction model. Therefore, after clustering, the sample data need to be filtered. In this paper, the Grubbs method was used to screen out the abnormal data in the data sample. The Grubbs method is mainly used to detect outliers in univariate data sets with normal distribution characteristics. Compared with other test methods, the Grubbs method is accurate and more sensitive to the test of outliers. However, it can only detect single-dimensional data and cannot accurately output normal intervals. Its judgment mechanism is to eliminate outliers one by one, so each outlier must be calculated separately. The whole step is only suitable for small-batch data, and the data are required to obey normal distribution or near-normal distribution [13,14].

## 3. Thermal Load Prediction Model of Proportional Solenoid Valve

### 3.1. Kalman Filtering Algorithm

The Kalman filtering (KF) algorithm is a recursive predictive filtering algorithm. The algorithm involves filtering and also involves the prediction of the data at the next moment [15]. KF is described by a series of recursive mathematical formulas. It provides an efficient and computable method to estimate the state of the process and minimize the mean square error of the estimation. KF can estimate the past and current state of a signal, and even estimate the future state, even if the exact nature of the model is not known. KF can also be considered as a data fusion algorithm. It was proposed more than 50 years ago and is one of the most widely used data fusion algorithms today. The advantages of KF are attributed to its small computational requirements, elegant recursive properties, and state as an optimal estimator for one-dimensional linear systems with Gaussian error statistics. KF can only reduce the impact of measurement noise with a mean of 0. As long as the noise expectation is 0, then no matter how large the variance is, as long as the number of iterations is enough, the effect is good. On the contrary, if the noise expectation is not 0, then the estimated value is still deviated from the actual value. Therefore, it is very suitable for solving real-time problems and embedded systems [16].

The KF algorithm uses the prior prediction value of the system state at the previous moment, the observation value at the current moment, the process noise, and the observation noise of the system to update the optimal estimation value of the system state [17,18,19]. The KF algorithm can rely on the measured value to continuously correct the estimated value with the iterative process, and has a good follow–up to the data. The system state model and observation model described by the KF algorithm are as shown in Equations (4) and (5):

(4) $$x_{t}=Ax_{t-1}+w_{t}$$

(5) $$y_{t}=Hx_{t}+v_{t}$$

In Formulas (4) and (5), *x_t_* denotes the system state of the target at time *t*, *x_t−_*_1_ denotes the system state of the target at time *t*−1, *A* denotes the parameter matrix of the state model, *w_t_* denotes the Gaussian white noise of the system process at time *t*, *y_t_* denotes the observed value of the target at time *t*, *H* denotes the parameter matrix of the observation model, and *v_t_* denotes the observed Gaussian white noise at time *t*.

### 3.2. Thermal Load Prediction Model

It is predicted that the original sample data will be automatically collected in the field test in a certain period, and can be directly used after conversion and preprocessing. There are two main sources of the thermal load of the solenoid valve. One is that the current in the coil of the proportional solenoid valve works on the copper conductor, and the other is that the magnetic field drives the armature displacement to work during the opening and closing process of the solenoid valve spool. Considering the large time domain of excavator operation and standby, the thermal load is mainly the work performed by the current in the coil on the copper conductor and dissipated to the surrounding environment in the form of heat. The relationship between thermal load and current is expressed by Equation (6):

(6) $$W=P\cdot t=\int_{0}^{t}I^{2}(t)\cdot R\cdot dt$$

In Equation (6), *W* is the thermal load, *P* is the power, *I* is the magnetic saturation current at the steady state of temperature, and *t* is the time.

It can be seen that the thermal load *W* has a linear relationship with the power *I*^2^*(t)·R*. Based on the linear positive correlation characteristics of the input and output of the solenoid valve, the approximate equivalent matching of the input and output relationship of the solenoid valve were obtained via the field test and the independent test, respectively. Taking the heating power of the proportional solenoid valve during the field test of the excavator as the object, it was used as the system state parameter *x_t_*, and the thermal load acting on the proportional solenoid valve during the field test was used as the observation parameter *y_t_*. Considering the difficulty of obtaining the observation parameters, the observation parameters were replaced by the heat load converted from the direct measurement value of the coil current in the independent test, and the thermal load was used as the reference value for comparison with the predicted value. The state parameters and observation parameters were superimposed by Gaussian white noise. Based on the KF algorithm, the state tracking and prediction of solenoid valve power and thermal load can be expressed.

Assuming that the influence of the transient moment of the excavator tooling switching on the system state was ignored, the system state remained stable under the operation of several specific tooling modes, and the matrix *A* in the recursive equations of the KF model was simplified to scalar 1. Since both power and thermal load are scalar data, the observation matrix *H* was simplified to scalar data according to the definition of the KF observation model. Therefore, the thermal load prediction model of the proportional solenoid valve running in the specific working mode of the excavator can be expressed as

(7) $$x_{t}^{*}=x_{t-1}^{\prime }$$

(8) $$P_{t}^{*}=P_{t-1}^{\prime }+Q$$

(9) $$k_{t}=P_{t}^{*}H{\left(H^{2}P_{t}^{*}+R\right)}^{-1}$$

(10) $$x_{t}^{\prime }=x_{t}^{*}+k_{t}(y_{t}-Hx_{t}^{*})$$

(11) $$P_{t}^{\prime }=([1-k_{t}H])P_{t}^{*}$$

In Equations (7)–(11), x_t_^*^ represents the heat load prediction value at time *t*, *x^t’^* represents the thermal load optimization estimation value, *P_t_^’^* represents the thermal load prediction value covariance, *k_t_* represents the KF gain, *y_t_* represents the power sampling value at time *t*, the initial state of the average thermal load under a specific working mode is represented by *P*_0′_, *H* represents the power–thermal load observation relationship, *Q* represents the heat load noise covariance, and *R* represents the power noise covariance.

### 3.3. Parameter Determination Method of Prediction Model

KF constantly uses new information to correct the estimated value in the recursive process. As the time series increases, the influence of the initial system state value and its covariance setting on the subsequent state will attenuate to nearly zero, so it can be set to zero. The values of *H*, *Q*, and *R* can be calculated with statistical methods from thermal load and power history samples. Under the condition of superimposed Gaussian white noise, the power measurement data and thermal load prediction data of the solenoid valve obey the normal distribution, and the relevant parameters can be determined with the unbiased estimation of the historical samples.

It is assumed that the existing data samples obeying normal distribution with a total amount of *N* are divided into *M* sample sets. In the *M* sample set, the number of samples in the sample set *i* is recorded as *N_i_* (*i =* 1,2,3,..., *M*), and the individual in the sample set is recorded as *X_i,k_* (*k =* 1,2,3,..., *Ni*). The arithmetic mean and standard deviation of sample set *i* can be represented by Equations (12) and (13), respectively:

(12) $$X_{i}=\frac{\sum_{k=1}^{N_{i}}X_{i,k}}{N_{i}}$$

(13) $$S_{i}^{2}=\frac{\sum_{k=1}^{N_{i}}{\left(X_{i,k}-X_{i}\right)}^{2}}{N_{i}-1}$$

According to the theory of mathematical statistics, the unbiased estimates of the normal distribution mean and covariance based on the sample can be expressed by Equations (14) and (15):

(14) $$\mu =\frac{\sum_{i=1}^{M}X_{i}}{M}$$

(15) $$\mu =\frac{\sum_{i=1}^{M}X_{i}}{M}$$

Furthermore, *R* is approximated as *σ*_2_ based on power samples, and *x*_0′_ and *Q* are approximated as *μ* and *σ*^2^ based on thermal load samples, respectively. Based on the definition of the KF observation model and the assumption that *v_t_* is Gaussian white noise, *H* is approximated as the ratio of *μ* value of power samples to *μ* value of thermal load samples.

## 4. Verification of Thermal Load Forecasting

### 4.1. Validation Method and Prediction Model Parameter Calculation

When the excavator is running in a specific working mode, the working mode of the proportional solenoid valve is divided into five categories, and the corresponding thermal load modes are marked separately. The model verification process is as follows:

(1) The historical data of proportional solenoid valve power corresponding to different working modes under excavator compound operation in field test is extracted.
(2) Collect the thermal load data of the solenoid valve corresponding to the specific single-action working mode.
(3) The power-thermal load historical data sequence is clustered, screened, and unbiasedly estimated to obtain the thermal load prediction model parameters under different working modes.
(4) Using the model parameters determined in step (3) to configure the KF thermal load prediction model, the thermal load prediction calculation of the installed solenoid valve is carried out.

**Figure 8:**
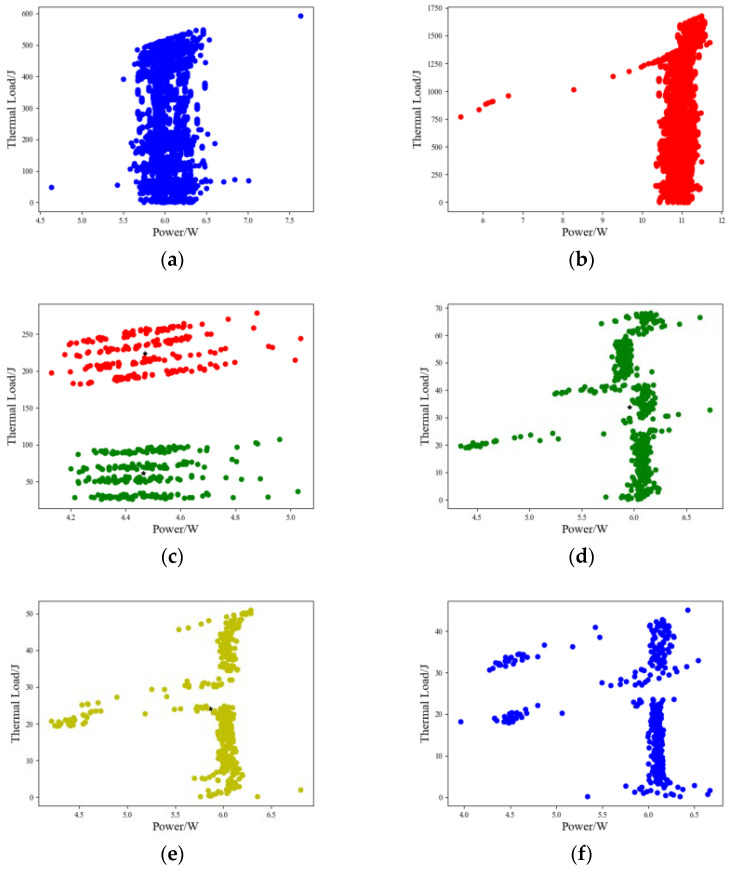
Single-action operation power–thermal load sample k-means clustering. (**a**) Low-speed walking, (**b**) high-speed walking, (**c**) 90° rotation, (**d**) boom, (**e**) bucket rod, and (**f**) bucket.

Based on the historical samples of the power and thermal load measured data of the proportional solenoid valve, the aforementioned k-means clustering algorithm was used for clustering. The clustering calculation was implemented using the k-means algorithm written in Python language. The clustering results are shown in Figure 8.

**Figure 9:**
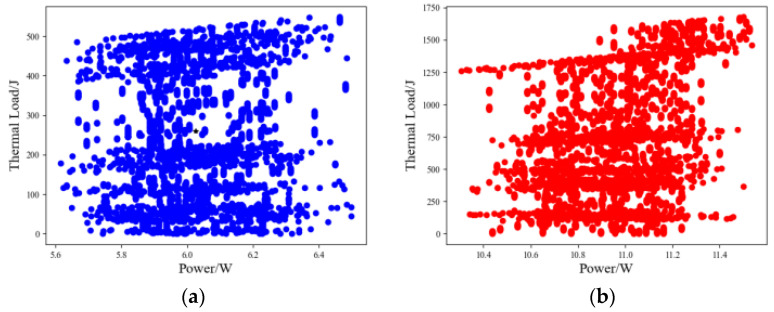
Sample clustering after Grubbs filtering. (**a**) Low-speed walking, (**b**) high-speed walking.

The clustering effects of low-speed and high-speed walking and rotary operation samples were more significant. The rotary operation clustering samples contained original data sampling in two time dimensions. The boom, bucket rod, and bucket clustering samples contained original data sampling in two time dimensions of detour and return. Here, based on the clustering sample data of walking operations, the Grubbs method with a significance level of 0.05 was further used to perform outlier analysis of abnormal data and perform screening processing. The processed sample clustering results are shown in Figure 9.

Unbiased estimation was implemented based on the clustering samples after abnormal data screening. These and later prediction calculations only took the low-speed walking operation mode as an example for detailed analysis. In Table 1, the parameters of the KF thermal load prediction model corresponding to the low-speed walking operation mode obtained via unbiased estimation are given.

### 4.2. Model Validation and Result Test

**Figure 10:**
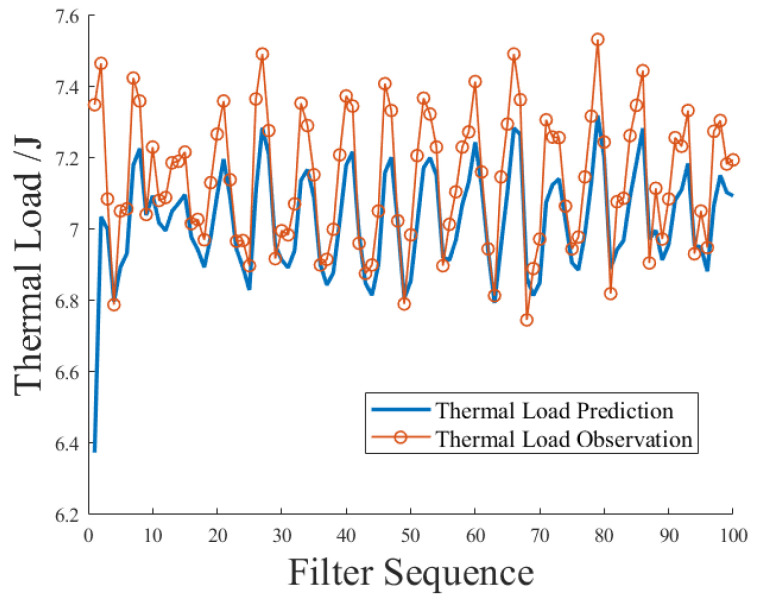
Synchronous comparison between predicted value and observed value of thermal load.

The KF thermal load prediction model was deployed by selecting the model parameters corresponding to the low-speed walking operation mode, and the heat load prediction operation was carried out based on the power measurement data in the field test. Figure 10 shows the synchronous comparison between the predicted heat load and the observed heat load. Here, only the first 100 sets of values in the actual prediction were intercepted for the convenience of the article.

**Figure 11:**
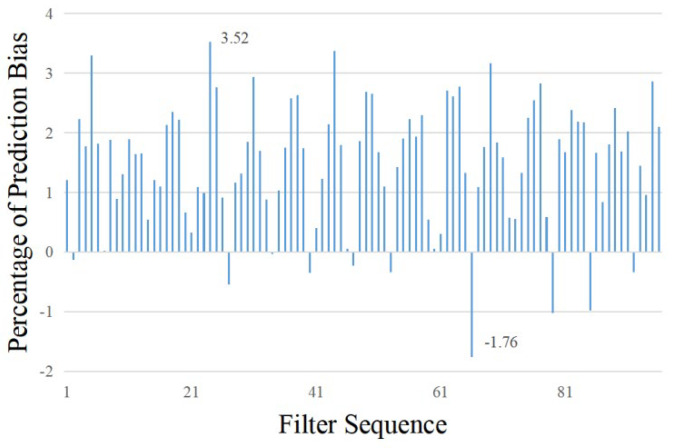
Percentage of thermal load prediction deviation.

The thermal load deviation is defined as the difference between the observed value and the predicted value of the heat load, and the percentage of the thermal load deviation is the percentage of the thermal load deviation and the observed value of the heat load. Figure 11 shows the synchronous comparison process from another perspective in the form of the percentage of energy consumption deviation. Consider that in the recursive process of KF, with the increase in time series, the influence of the initial system state value and its covariance setting on the subsequent state was attenuated to nearly zero. We filtered out the predicted maximum deviation value which was greatly affected by the initial system state value and analyzed the remaining predicted value sequence. The results show that the mean value of the corresponding thermal load prediction deviation percentage in the low-speed walking operation mode was 1.46%, and the maximum and minimum values were 3.52% and –1.76%.

**Figure 12:**
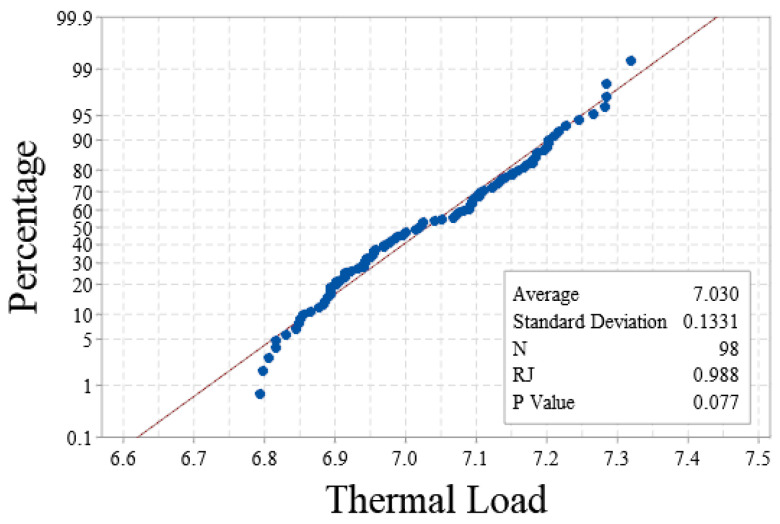
Normality test of thermal load prediction value of low-speed walking operation.

Figure 12 shows the normality test results of the predicted value of the proportional solenoid valve thermal load based on the Ryan–Joiner method [20] corresponding to the low-speed walking operation mode. The results show that the *RJ* value was 0.988, which is very close to 1, and the *p* value was 0.077, which is greater than 0.05, which meets the requirement of a 0.95 confidence level; that is, it conforms to the normal distribution. The Anderson–Darling method and Kolmogorov–Smirnov method were used to test the normality of the predicted values. Considering that the process data to be analyzed may have abnormal values, the Ryan–Joiner test is the most appropriate. The Anderson–Darling test is more appropriate for the possibility of data drift in the data acquisition process, resulting in a bimodal distribution. The Kolmogorov–Smirnov detection effect is not as good as the other two in different cases [21,22].

### 4.3. Compared with the Prediction Results of Other Models

In order to test the prediction effect of the KF thermal load prediction model under normal conditions, based on the stationary time series characteristics of the model processing data and taking the low-speed walking operation of the excavator as an example, the thermal load prediction effects of the autoregressive integrated moving average (ARIMA) model and the moving average (MA) model were tested with the same data samples to be tested as a comparison [23,24,25]. The predicted value of the MA model thermal load is *W_m_(n)*, the predicted value of the ARIMA model heat load is *W_a_(n)*, and the predicted value of the KF thermal load is *W_kf_*. The average percentage error of the thermal load deviation is MAPE, the average absolute error of the thermal load deviation is MAD, and the root mean square error of the thermal load deviation is RMSE. Table 2 is a statistical feature comparison of the predicted value sequence of the three thermal load models. From the statistical results of Table 2, it can be seen that the prediction effect of the KF thermal load prediction model showed the optimal level of the three.

**Figure 13:**
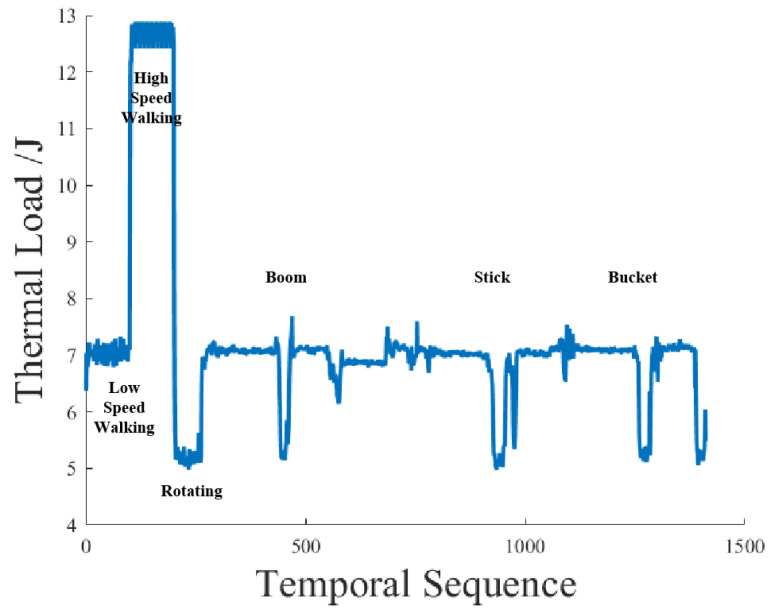
Thermal load spectrum of the proportional solenoid valve of excavator main pump.

Based on the models and methods used in this paper, as shown in Figure 13, the overall prediction calculation of the samples obtained based on the test is given, and the thermal load spectrum of the proportional solenoid valve corresponding to the different operating modes of the excavator is obtained. In the process of excavator compound operation, the thermal load of the proportional solenoid valve is the regular superposition of each sub-spectrum data point in the load spectrum.

## 5. The Application Test of the Prediction Model under Specific Samples

Performing the application test under specific samples is helpful for verifying the fault tolerance and adaptive ability of the prediction model. The test content had two main aims. One was to simulate and test the application of the thermal load prediction model in the automatic filling of the missing data and the automatic repair of the abnormal data based on the experimental data obtained in the model verification process. The other was to simulate and test the application of the thermal load prediction model in the adaptive detection of abnormal fluctuations of the thermal load measurement data based on the experimental data obtained in the model verification process. This chapter takes the sample data corresponding to the low-speed walking operation mode as an example for test verification.

### 5.1. Application Test of Data Missing Filling and Data Anomaly Repair

**Figure 14:**
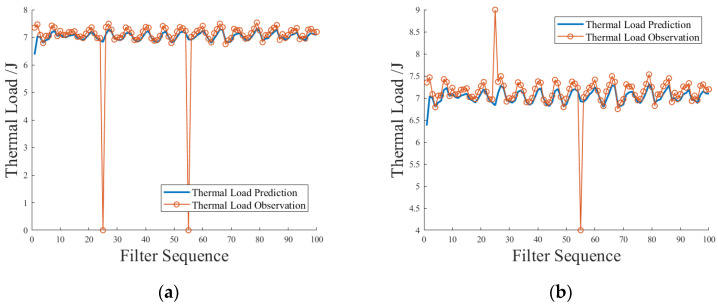
Simulation test of automatic missing filling and data anomaly repair for thermal load measurement value via thermal load prediction value. (**a**) Automatic missing filling, (**b**) data anomaly repair.

The thermal load prediction value x_t_^*^ of the solenoid valve thermal load prediction model at time t can be combined with the power observation data at that time to obtain the optimal estimation value *x_t_^’^* of the thermal load at time *t*. *x_t_^’^* is numerically equal to the thermal load prediction value at the next moment. It can be used as a missing fill when the thermal load measurement data are lost at the next moment. It can also be used as an adaptive measure of whether the thermal load measurement data are abnormal at the current moment. Figure 14a,b show the simulation scenarios of automatic missing filling and data anomaly repair of thermal load prediction value to thermal load measurement value, respectively. In the simulation scenario with missing data, the measured data of the thermal load with a value of 6.8984 were set to 0.0 to simulate the loss. The label in the figure is the predicted value of the thermal load corresponding to the lost thermal load measurement data. The percentage of thermal load deviation at the automatic filling is 0.98% and –0.33%. In the data anomaly simulation scenario, the measured data of thermal load with a value of 6.8984 were set to 9.0 and 4.0 to simulate the data anomaly. The label in the figure is the predicted value of the thermal load corresponding to the measured data of the abnormal thermal load. The percentage of thermal load deviation of automatic repair is 1.02% and –0.53%. Through the analysis of the test results, it can be seen that the KF thermal load prediction model can better meet the application requirements of missing filling and data anomaly repair.

### 5.2. Application Test of Abnormal Adaptive Detection of Thermal Load Data

**Figure 15:**
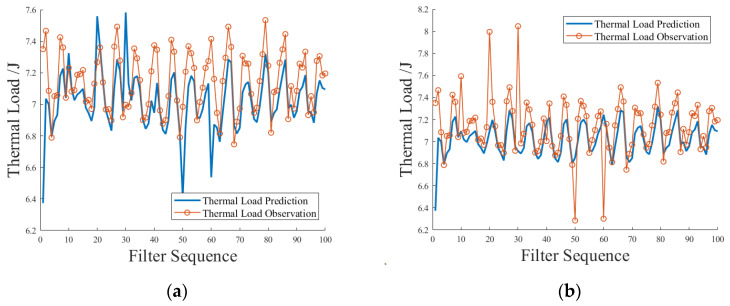
Simulation test of power measurement data fluctuation or thermal load measurement data fluctuation only. (**a**) Power measurement data fluctuation, (**b**) thermal load measurement data fluctuation.

The adaptive detection of thermal load measurement data anomalies is referred to as thermal load anomaly detection. Thermal load anomaly detection does not use a fixed value, but uses the percentage of thermal load deviation defined above as a measure of whether the thermal load measurement data fluctuate abnormally. The test scheme set the percentage of thermal load deviation more than ±10% as abnormal, and within ±10% as normal. Since the power measurement data used in the thermal load prediction model may also fluctuate, the influence of the fluctuation of the power measurement value on the detection effect was considered during the test. The test scheme randomly selects six moments to scale the size of the experimental measurement data by 5%, 10%, and 15% to simulate the different situations of normal fluctuation, critical fluctuation, and abnormal fluctuation of the measurement data. The test results and explanations of the simulated scene are as follows. Figure 15a shows the simulation scenario where only the power measurement data fluctuate. It can be seen from the figure that due to the feedback regulation of Kalman gain, the critical fluctuation and partial abnormal fluctuation of power measurement data do not lead to a false alarm in thermal load anomaly detection. The thermal load anomaly detection application has good fault tolerance for the fluctuation of power measurement data. Figure 15b shows a simulation scenario where only the thermal load measurement data fluctuate. It can be seen from the figure that the normal fluctuation, critical fluctuation, and abnormal fluctuation of the thermal load measurement data were successfully identified, and the application of thermal load anomaly detection achieved the expected results.

**Figure 16:**
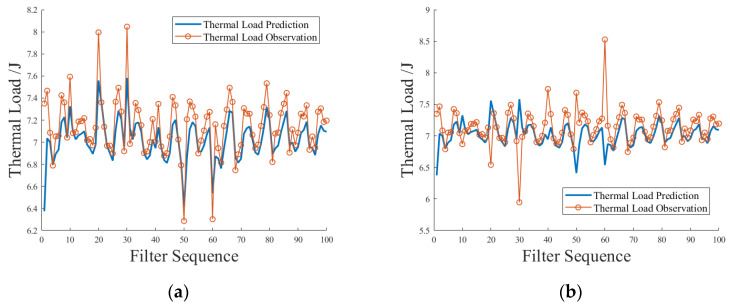
Simulation test of power measurement data and thermal load measurement data fluctuating in the same direction or opposite direction at the same time. (**a**) In the same direction, (**b**) in opposite direction.

Figure 16a shows a simulation scenario in which the power measurement data and the thermal load measurement data fluctuate in the same direction at the same time. It can be seen from the figure that due to the linear relationship between power and thermal load, the sensitivity of the thermal load anomaly detection application was suppressed, and a missed alarm occurred. The critical fluctuation and abnormal fluctuation of thermal load measurement data were wrongly identified as normal fluctuation. Figure 16b shows a simulation scenario in which the power measurement data and the thermal load measurement data simultaneously fluctuate in the opposite direction. From the graph, it can be seen that the sensitivity of the thermal load anomaly detection application was magnified, the critical fluctuation and abnormal fluctuation were easier to identify, and the possibility of normal fluctuation being misidentified became larger.

Table 3 shows the percentage of deviation between the predicted and observed values of thermal load in four different scenarios. Considering the random interference and probability statistics that may be encountered in the actual working conditions, power and thermal load data acquisition, and measurement of excavator operation, the possibility of the simultaneous fluctuation of power and thermal load measurement data is much lower than that of single thermal load measurement data. Under extreme operating conditions such as high humidity and extreme cold, positive and negative fluctuations in power and thermal load measurement data may be briefly observed. The particularity of the conditions determines the non-applicability of the prediction theory. The analysis and prediction work needs to be completed under the constraints of specific conditional characteristics. Based on the above test results, it can be considered that the application of thermal load anomaly detection based on the KF thermal load prediction model is feasible.

## 6. Conclusions

This paper discusses a thermal load prediction method for proportional solenoid valves which can be used in practical engineering. In order to achieve this goal, based on the Kalman filter algorithm framework, the noise parameters of the prediction model were determined via the statistical calculation of historical samples, and the thermal load data were predicted based on the power measurement values. In order to better realize this prediction, the original data samples for a field test and independent test of the non-installed solenoid valve were designed. The k-means clustering method was proposed to identify and distinguish each single-action operation mode in the excavator composite operation and provide data samples corresponding to different working modes for the thermal load prediction model that can better reflect the operation characteristics. The Grubbs method was used to screen out the outliers of the clustered samples to obtain better data samples to be predicted. The prediction results of thermal load show that the predicted value met the requirements of normal distribution well, and the percentage of prediction deviation could be effectively controlled within 5%. The model can meet most engineering application scenarios. Compared with the thermal load prediction results of other prediction models, the KF prediction model performed best in the three aspects of average percentage error, average absolute error, and root mean square error of prediction deviation. Through the application test of the prediction model under specific samples, it was verified that the model has certain potential abilities in missing data filling, data anomaly repair, and anomaly adaptive detection. It can be seen that the KF thermal load prediction model has high prediction accuracy and small deviation. The improvement and optimization of the KF algorithm model help to improve the accuracy and applicability of the prediction method. The construction method of the thermal load spectrum can provide some theoretical support for the study of thermal reliability and the prediction of the remaining useful life of proportional solenoid valves and similar electromechanical products.

Under typical working conditions, the action process of a proportional solenoid valve with a specific structure is similar to that of a proportional solenoid valve with other working conditions and different structures under single and compound working conditions. When considering different characteristics, the thermal load prediction results differ. Methods of combining the energy consumption and load of a whole machine to connect the action process of a proportional solenoid valve with working conditions require further study. Based on the existing research data, it was shown that the characterization method for the thermal load prediction of a proportional solenoid valve is more flexible and includes uncertainty. The selection of more advanced prediction and analysis algorithms has a positive effect on the reliability of thermal load prediction results. The future research results based on the research content of this article are a durability analysis model of proportional solenoid valves and an online thermal fault monitoring platform of construction machinery and vehicle components.

## Figures and Tables

**Table 1:** Parameters of KF thermal load prediction model corresponding to low-speed walking operation mode.

Parameters	*R*	*H*	*X*_0′_	*Q*
Low-speed walking	0.1622	0.8460	7.1218	0.1910

**Table 2:** Comparison of statistical characteristics of prediction results of three prediction models.

Statistics	*W_k_f*	*W_m_(n)*	*W_a_(n)*
MAPE	1.4626	2.3492	3.1248
MAD	0.1269	0.1645	0.1732
RMSE	0.1975	0.2067	0.2613

**Table 3:** Percentage of deviation between the predicted and observed values of thermal load in four different scenarios.

Fluctuation Form	Only Power Fluctuation	Only Thermal Load Fluctuation	Power and Thermal Load Fluctuate in the Same Direction	Power and Thermal Load Fluctuate in the Reverse Direction
5%	−1.29	6.56	3.53	−6.63
10%	−4.01	11.23	5.45	−15.57
15%	−8.37	14.06	5.76	−27.50
−5%	5.81	−2.49	0.86	10.30
−10%	8.22	−9.04	−1.97	16.57
−15%	11.83	−14.95	−3.72	23.34

## Data Availability

Data are contained within the article.
